# Seed Dormancy in Arabidopsis Requires Self-Binding Ability of DOG1 Protein and the Presence of Multiple Isoforms Generated by Alternative Splicing

**DOI:** 10.1371/journal.pgen.1005737

**Published:** 2015-12-18

**Authors:** Kazumi Nakabayashi, Melanie Bartsch, Jia Ding, Wim J. J. Soppe

**Affiliations:** Department of Plant Breeding and Genetics, Max Planck Institute for Plant Breeding Research, Cologne, Germany; University of York, UNITED KINGDOM

## Abstract

The Arabidopsis protein DELAY OF GERMINATION 1 (DOG1) is a key regulator of seed dormancy, which is a life history trait that determines the timing of seedling emergence. The amount of DOG1 protein in freshly harvested seeds determines their dormancy level. DOG1 has been identified as a major dormancy QTL and variation in *DOG1* transcript levels between accessions contributes to natural variation for seed dormancy. The *DOG1* gene is alternatively spliced. Alternative splicing increases the transcriptome and proteome diversity in higher eukaryotes by producing transcripts that encode for proteins with altered or lost function. It can also generate tissue specific transcripts or affect mRNA stability. Here we suggest a different role for alternative splicing of the *DOG1* gene. *DOG1* produces five transcript variants encoding three protein isoforms. Transgenic *dog1* mutant seeds expressing single *DOG1* transcript variants from the endogenous *DOG1* promoter did not complement because they were non-dormant and lacked DOG1 protein. However, transgenic plants overexpressing single DOG1 variants from the 35S promoter could accumulate protein and showed complementation. Simultaneous expression of two or more *DOG1* transcript variants from the endogenous *DOG1* promoter also led to increased dormancy levels and accumulation of DOG1 protein. This suggests that single isoforms are functional, but require the presence of additional isoforms to prevent protein degradation. Subsequently, we found that the DOG1 protein can bind to itself and that this binding is required for DOG1 function but not for protein accumulation. Natural variation for DOG1 binding efficiency was observed among Arabidopsis accessions and contributes to variation in seed dormancy.

## Author Summary

The Arabidopsis protein DELAY OF GERMINATION 1 (DOG1) is an important regulator of seed dormancy and controls the timing of seedling emergence. The amount of DOG1 protein in mature seeds correlates with their dormancy level. It has been demonstrated that DOG1 is an important contributor to natural variation for seed dormancy and Arabidopsis accessions vary in their DOG1 levels. In this study we showed that the *DOG1* gene produces five transcript variants encoding three protein isoforms. Transgenic *dog1* mutant seeds expressing single *DOG1* transcript variants lack dormancy and do not accumulate DOG1 protein. Simultaneous expression of two or more *DOG1* transcript variants encoding different isoforms, however, leads to the accumulation of DOG1 protein and increased seed dormancy. DOG1 protein can bind to itself and Arabidopsis seeds that contain a DOG1 variant unable to bind show reduced seed dormancy. We observed variation for DOG1 binding efficiency between Arabidopsis accessions, indicating a role for DOG1 self-binding in natural variation for seed dormancy.

## Introduction

Alternative splicing has an important role in the post-transcriptional regulation of higher eukaryotes, but it was long believed to be of minor significance in plants. During the last years consecutive reports demonstrated a steadily increasing percentage of alternatively spliced genes in plants. At the beginning of this century only 1.5% of the *Arabidopsis thaliana* genes were estimated being alternatively spliced [1]. Within one decade this fraction went up to 61% [2]. Alternative splicing can lead to different outcomes and produces transcripts that code for proteins with altered or lost function. It can also lead to tissue specific transcripts or affect mRNA stability and turnover via nonsense-mediated decay [3,4]. The regulation and function of alternative splicing in plants is still largely unexplored but several examples have demonstrated its functional importance in various processes like photosynthesis, defence responses, the circadian clock, hormone signalling, flowering time, and metabolism [5,6,7,8]. A few examples have shown a role of alternative splicing during seed development and germination. The central regulator of seed maturation, *ABSCISIC ACID INSENSITIVE 3* (*ABI3*), has been cloned in Arabidopsis over 20 years ago [9], but it was only recently found that this gene is alternatively spliced in a developmentally regulated fashion [10]. The *PHYTOCHROME INTERACTING FACTOR 6* gene is also alternatively spliced during seed development and one of its two splice forms, *PIF6-β* influences germination potential [11].

The timing of seed germination determines the seasonal environmental conditions that a plant encounters during its life and thereby its growth and reproductive success. Seed germination is regulated by dormancy, which is defined as the incapacity of a viable seed to germinate under favourable conditions [12]. Seed dormancy is induced during seed maturation and released by dry storage of seeds (after-ripening) or imbibition at low temperatures (stratification) [13], and is regulated by environmental and endogenous factors. Extensive research on seed dormancy in several plant species, including Arabidopsis, has revealed the requirement of the plant hormone abscisic acid (ABA) to induce dormancy during seed maturation, whereas gibberellins (GAs) are required for germination [13,14]. In addition, mutant analyses identified a number of seed dormancy regulators. Apart from factors involved in hormone metabolism and seed maturation, these included several chromatin modifiers and transcriptional regulators [15].

The Arabidopsis gene *DELAY OF GERMINATION 1* (*DOG1*) is a master regulator of seed dormancy acting independent of ABA. *DOG1* was first identified as a major Quantitative Trait Locus (QTL) for seed dormancy [16]. Mutations in the *DOG1* gene lead to a complete absence of dormancy. *DOG1* shows a seed-specific expression pattern and encodes a protein with unknown function. Its transcript and protein abundances in freshly harvested seeds highly correlate with dormancy levels [17,18]. This correlation has been observed under both lab and natural conditions. Environmental conditions that enhance seed dormancy, such as low temperatures during seed maturation, are associated with enhanced *DOG1* transcript levels [18,19,20]. Arabidopsis accessions from the south of Europe are in general more dormant and show higher *DOG1* transcript levels compared to northern accessions [19]. Interestingly, *DOG1* transcript levels also showed the highest correlation among a set of seed dormancy genes with changes in dormancy in buried seeds in the field [21]. During after-ripening DOG1 protein remains stable but loses its activity due to unknown post-translational modifications. Therefore, DOG1 is likely to be part of a timing mechanism for the release of seed dormancy [18]. The DOG1 protein belongs to a small family in Arabidopsis that is conserved in plants. Several *DOG1* homologues have been shown to be functionally conserved and are able to enhance seed dormancy in *Lepidium sativum* [22] and *Triticum aestivum* [23].

We are only starting to understand the regulation of DOG1 at the protein level and its transcriptional regulation remains to be further investigated. *DOG1* transcript levels are enhanced by the TFIIS transcription elongation factor [24,25] and by histone monoubiquitination [26]. *DOG1* is alternatively spliced and four splicing variants encoding three different isoforms have been reported [17]. It has recently been shown that the spliceosome disassembly factor NTR1 is required for proper transcript levels and splicing of *DOG1* [27].

We have identified a fifth splicing variant of *DOG1* that constitutes the majority of its transcripts. Here we show that the accumulation of DOG1 protein requires alternative splicing because single DOG1 protein isoforms are not able to accumulate efficiently in the seed. The DOG1 protein can bind to itself and this self-binding is required for full DOG1 function. Variation in self-binding ability of DOG1 exists in nature and contributes to variation in seed dormancy levels between Arabidopsis accessions.

## Results

### Quantification of DOG1 transcripts during seed maturation

**Fig 1:**
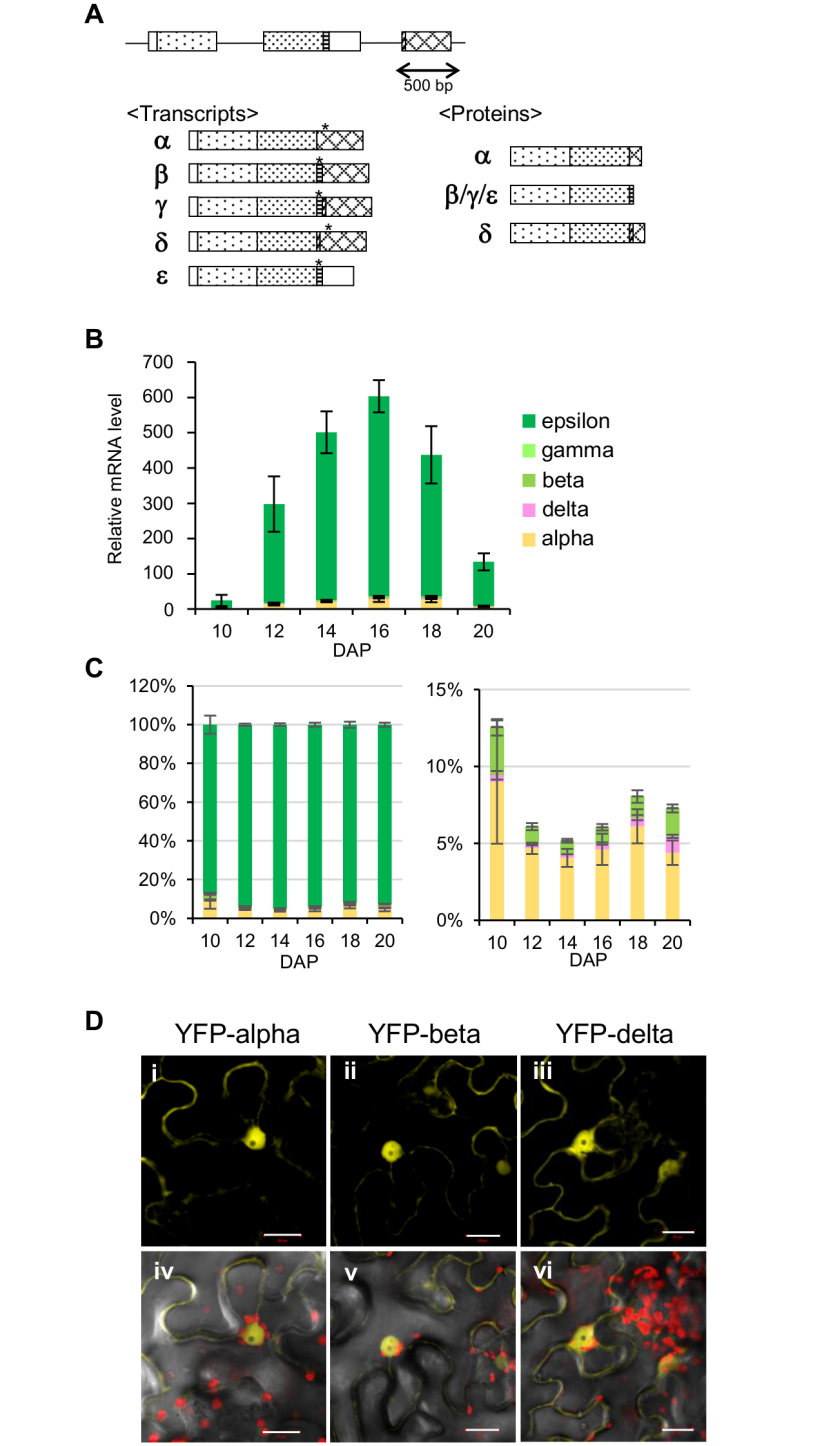
Five alternative splicing variants of *DOG1* encode three protein isoforms that are localised in the nucleus. (**A**) Schematic representation of transcript variants and protein isoforms. The genomic structure is presented at the top. Exons are shown as boxes, and introns, the partial promoter and downstream regions as lines; asterisk represents STOP codon. (**B**) qRT-PCR analysis of *DOG1* splicing variants during seed maturation of NIL DOG1. The mRNA level of each *DOG1* variant was normalised to the *ACT8* mRNA level. Error bars represent the S.E.M. of at least three biological replicates. DAP, days after pollination. (**C**) The relative ratios of the *DOG1* splicing variants during seed maturation, calculated from the data in (B). The right panel is a magnified image of the bottom of the graph in the left. For clarity, the epsilon variant is not shown in the right panel. Error bars represent S.E.M. DAP, days after pollination. The data normalised to the second reference gene *HBT* for (B) and (C) are shown in S1 Fig. (**D**) Subcellular localisation of DOG1 isoforms fused to YFP transiently expressed in *Nicotiana benthamiana* leaves. YFP fluorescence (i–iii), merged image of YFP fluorescence, autofluorescence of chlorophyll, and transmission (iv–vi). Scale bars = 20 μm.

The Arabidopsis *DOG1* gene contains three exons. Its second intron is alternatively spliced and shows both alternative 3’ and alternative 5’ splice site selection, leading to four different transcripts *DOG1-α*, *DOG1-β*, *DOG1-γ*, and *DOG1-δ* [17]. We quantified the individual transcripts using specific primers. Comparison of total *DOG1* transcript levels with the combined levels of the four individual splicing forms indicated that the majority of *DOG1* transcript does not exist out of these four forms. Detailed analysis of the *DOG1* transcripts by 3’-RACE revealed the presence of a fifth splicing form encoding the same protein as *DOG1-β* and *DOG1-γ*, which was designated as *DOG1-ε*. This splicing form misses the complete third exon (Fig 1A).

The abundance of the five *DOG1* splicing variants was followed during seed maturation. As shown previously [17,18], *DOG1* expression peaks in the middle of the seed maturation phase and is reduced in fully mature dry seeds. The *DOG1-ε* transcript represents about 90–95% of the *DOG1* transcripts at the measured time-points (Fig 1B and S1A Fig). The ratio between the different splicing forms is fairly constant (Fig 1C and S1B Fig), although *DOG1-α* is relatively more abundant at the beginning of seed maturation. *DOG1-δ* is very low abundant, but increases at the end of seed maturation (Fig 1C and S1B Fig).

The alternative splicing of DOG1 only affects the C-terminal part of the protein. DOG1-β is the smallest isoform (consisting of 278 amino acids in the Landsberg *erecta* (L*er*) accession) and shares nearly the complete protein sequence with DOG1-α and DOG1-δ, apart from its last nine amino acids. The DOG1-α and DOG1-δ proteins are longer, respectively 292 and 303 amino acids in L*er*, and share their last 24 amino acids with each other (Fig 1A).

### All three DOG1 isoforms are located in the nucleus

The cellular localisation of the three DOG1 isoforms was analysed by transient expression of their N-terminal yellow fluorescent protein (YFP) fusion proteins in *Nicotiana benthamiana* leaves. All three fusion proteins were mainly detected in the nucleus similar as previously shown in transgenic Arabidopsis seeds containing YFP fused with the DOG1 genomic fragment (Fig 1D) [18]. These results were confirmed by transformation of Arabidopsis protoplasts with C-terminal YFP fusion proteins of the three DOG1 isoforms (S2 Fig). The presence of all three DOG1 protein isoforms in the nucleus suggests that they are able to meet each other.

### The accumulation of DOG1 protein requires the presence of multiple isoforms

**Fig 2:**
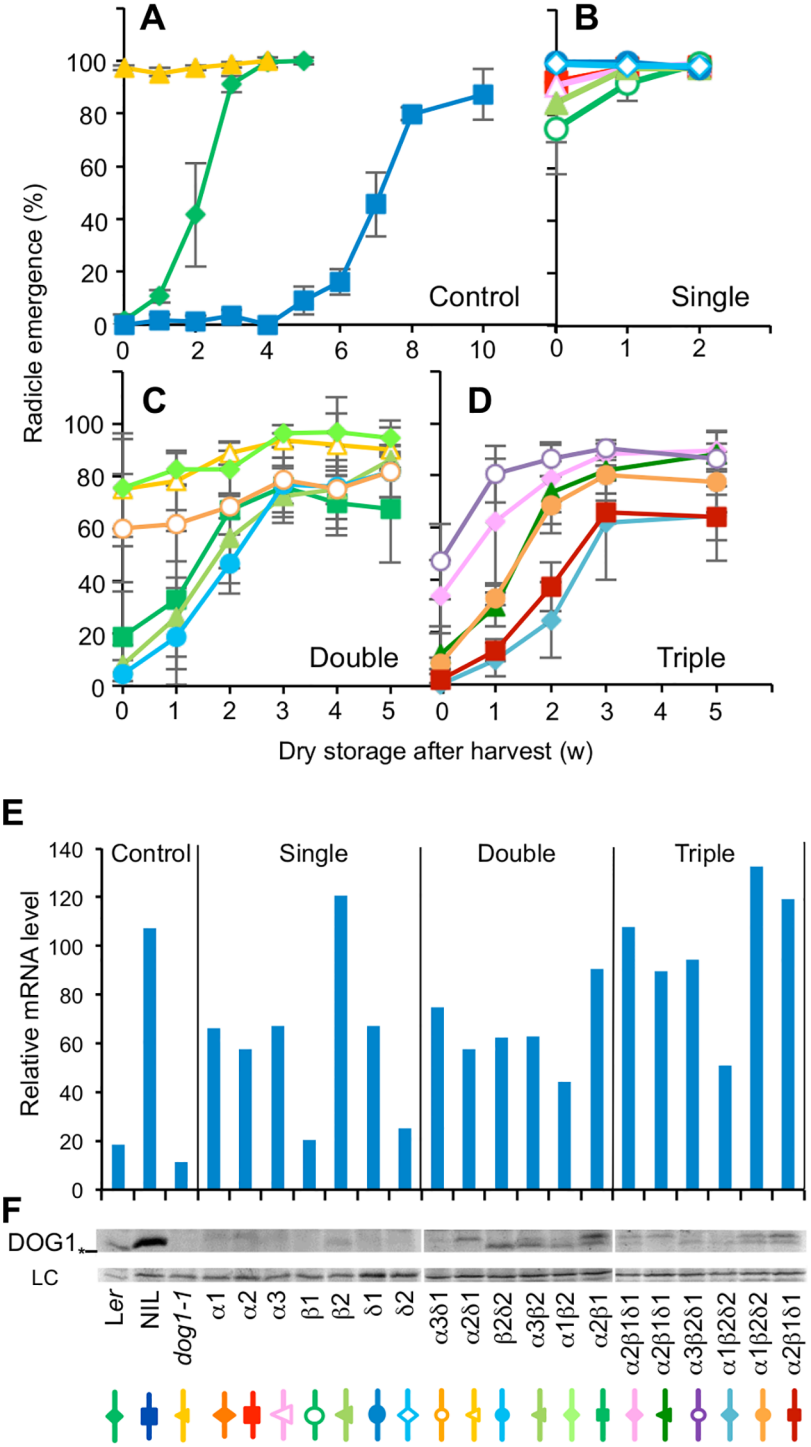
The accumulation of DOG1 requires the presence of multiple isoforms. Germination profiles of control lines (**A**), single (**B**), double (**C**) and triple (**D**) *pDOG1_Cvi*:*DOG1-α*, *DOG1-β*, and *DOG1-δ* transformants in *dog1-1* after different periods of dry storage. Error bars represent S.E.M. of at least three biological replicates. w, week. *DOG1* overall mRNA (**E**) and protein (**F**) levels in transgenic lines. (**E**) *DOG1* mRNA level was normalised to *ACT8* mRNA level. (**F**) The top panel shows DOG1 protein, and the bottom panel a nonspecific band around 60 kD that is used as loading control (LC) [18]. An asterisk on the left of the top panel shows molecular mass marker around 36 kD. The *dog1-1* mutant produces only truncated protein and serves as a negative control. Individual line names were indicated at the bottom for (E) and (F). The line colours beneath the transgenic line names correspond to the line colours in (A-D).

The Near Isogenic Line DOG1 (NIL DOG1) contains an introgression of a strong *DOG1* allele from the Cape Verde Islands (Cvi) accession in the L*er* background and has a high level of seed dormancy. In contrast, the *dog1-1* mutant (in NIL DOG1 background) does not produce full-length DOG1 protein and is nondormant (Fig 2A) [17]. The function of the three DOG1 protein isoforms was studied by complementation of the *dog1-1* mutant with single DOG1 isoforms driven by the native *DOG1_Cvi* promoter. Transgenic plants with single insertion events were obtained. None of these showed convincing functional complementation as their seed dormancy level was comparable to that of the *dog1-1* mutant. Only introduction of DOG1-β caused a very weak restoration of dormancy (Fig 2B). Therefore, single DOG1 isoforms appeared largely non-functional when expressed from a native *DOG1* promoter.

Subsequently, transgenic plants with single DOG1 isoforms were mutually crossed and F3 or F4 plants homozygous for two isoforms were selected for all possible combinations. Transgenic lines containing two DOG1 isoforms showed enhanced dormancy compared to lines containing a single DOG1 isoform. Although dormancy levels of these double transformants showed variation, we observed the tendency that plants containing a combination of DOG1-β with DOG1-α or DOG1-δ had higher dormancy levels than plants containing a combination of DOG1-α and DOG1-δ, which germinated already 70–80% directly after harvest (Fig 2C). The dormancy level of double transgenic plants containing DOG1-β as one of the two splicing forms was similar to that of the low dormant L*er* accession but much lower than NIL DOG1, which is the wild-type background of the *dog1-1* mutant. Finally, we obtained transgenic plants that contain all three DOG1 isoforms after further crossing and selection. Seeds of all these triple transgenic plants showed dormancy restoration and none of them had similar low dormancy levels as the double transgenic lines containing α and δ (Fig 2D).

Seed dormancy highly correlates with *DOG1* transcript and protein levels in freshly harvested seeds [18]. Therefore, transcript and protein levels were measured in the single, double and triple transgenic plants. Freshly harvested seeds of the transgenic lines showed some variation in their *DOG1* expression but were mostly in a similar range as those of NIL DOG1 (Fig 2E). This was to be expected because *DOG1* is transcribed from the same *DOG1*_Cvi promoter in NIL DOG1 and the transgenic lines. The transcript levels of the transgenes are expected to be slightly lower than the observed *DOG1* transcript levels, which include a low amount of transcript from the *dog1* mutant gene. Taken this into account, transcript levels in the transgenic lines still did not correlate with dormancy levels. Several of the transgenic lines with single DOG1 isoforms showed high *DOG1* expression levels, comparable to NIL DOG1, but were non-dormant. In contrast, dormancy correlated with DOG1 protein accumulation in these transgenic seeds (Fig 2B–2F). DOG1 protein was scarcely detectable in the transgenic lines containing single DOG1 isoforms, in accordance with their lack of seed dormancy. The double and triple transgenic lines accumulated varying levels of DOG1 protein that were mostly in the same range as those found in seeds of the L*er* accession, but much lower compared to the wild-type NIL DOG1. Interestingly, the double transgenic lines accumulated slightly higher levels of DOG1 protein than the triple, although they were less dormant. This suggests that the presence of three isoforms gives higher DOG1 activity in comparison to two isoforms. In the double and triple transformants that contain both DOG1-β and DOG1-α or DOG1-δ protein, two bands could be detected. In the non-transgenic controls, however, only the faster migrating band is visible, which corresponds to the DOG1-β protein. This is most likely due to the different ratio of the *DOG1* transcript variants between controls and transgenic lines. In the double and triple transformants the different *DOG1* transcript variants are expressed at similar levels, whereas in the wild type the transcripts that encode the DOG1-β protein are most abundant (Fig 1C). As DOG1 protein accumulates to higher levels in NIL DOG1 compared to the double and triple transgenic lines despite their comparable transcript levels, the ratio between the isoforms probably influences DOG1 protein accumulation.

### Single DOG1 isoforms can accumulate when highly overexpressed

**Fig 3:**
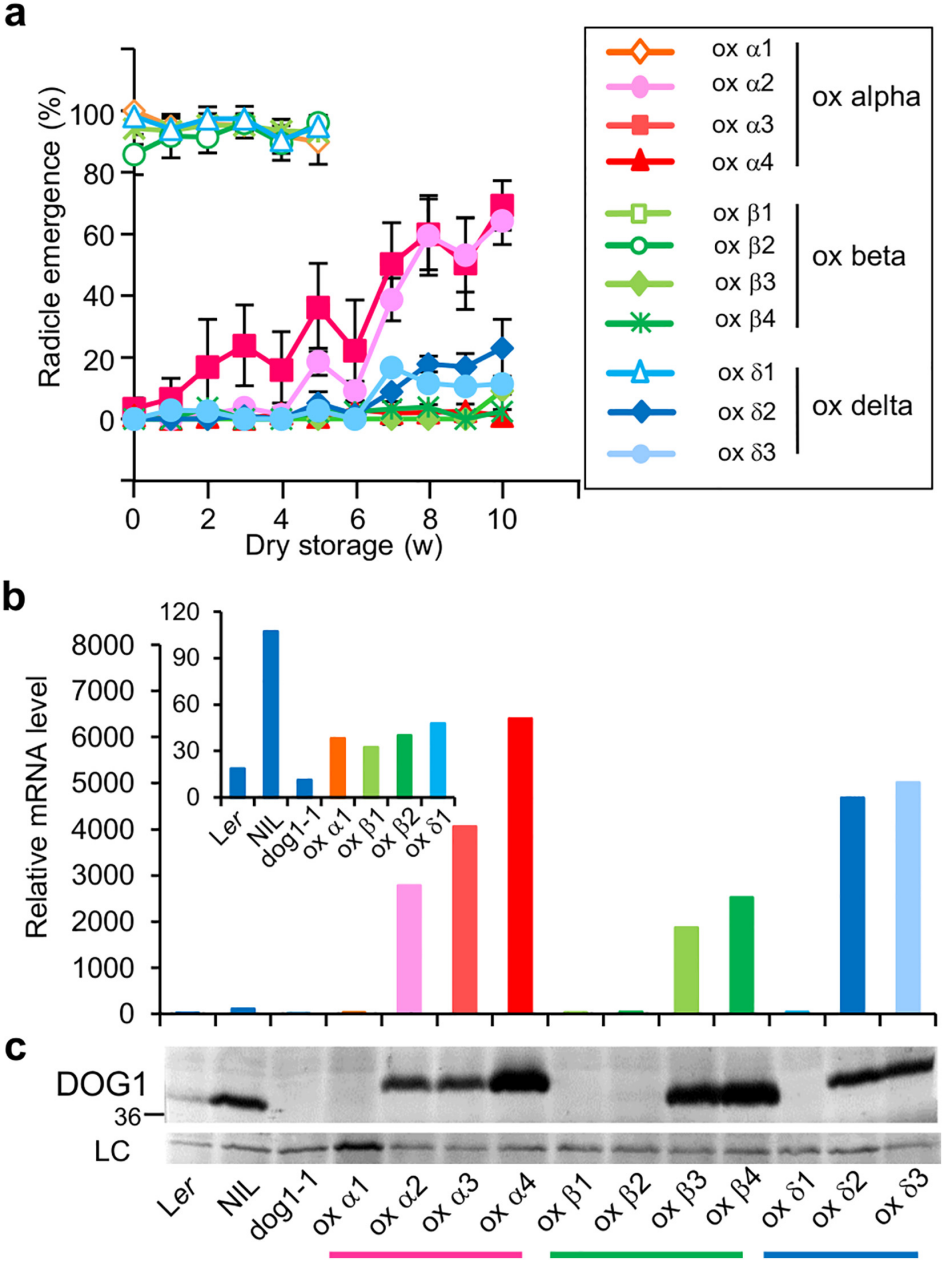
Single isoforms can induce dormancy when strongly overexpressed. (**A**) Germination profiles of *p35S*:*DOG1-α*, *DOG1-β*, and *DOG1-δ* transformants in *dog1-1* after different periods of dry storage. Error bars represent S.E.M. of at least three biological replicates. w, week. Germination profiles of the control lines are shown in Fig 2A. *DOG1* overall mRNA (**B**) and protein (**C**) levels in overexpression transformant lines. (**B**) *DOG1* mRNA levels were normalised to *ACT8* mRNA level. The top left graph shows the values for the controls and non-complementing transformant lines. (**C**) Details are described in the legend of Fig 2F.

We were interested whether an increase in the transcript level of single *DOG1* transcripts could lead to complementation of the *dog1* mutant. Three constructs with *DOG1-α*, *DOG1-β*, and *DOG1-δ* driven by the constitutive 35S promoter were separately transformed into *dog1-1* plants. About 10 to 40% of the obtained independent transformants for all three constructs showed high levels of seed dormancy (Fig 3A). Interestingly, the DOG1-β isoform was most effective in dormancy induction, followed by DOG1-δ. DOG1-α showed relatively lower dormancy levels. Overall, this experiment demonstrated that every DOG1 isoform is biochemically functional to induce seed dormancy.

Both complementing and non-complementing lines for all three constructs were selected for further analysis. Comparison of *DOG1* transcript levels between these lines showed a more than 100-fold difference. High levels of *DOG1* transcript were only detected in lines with high dormancy levels (Fig 3B). Consistent with the transcript levels, DOG1 protein could only be detected in the dormant transformants where it accumulated to even higher levels than in the NIL DOG1 control (Fig 3C). Taken together with the results from the complementation experiments using the native DOG1 promoter (Fig 2A–2F), these data suggest that all the single DOG1 isoforms are functional but unstable in the cell and can only accumulate when they have very high transcript levels.

### Self-binding of DOG1 enhances seed dormancy

**Fig 4:**
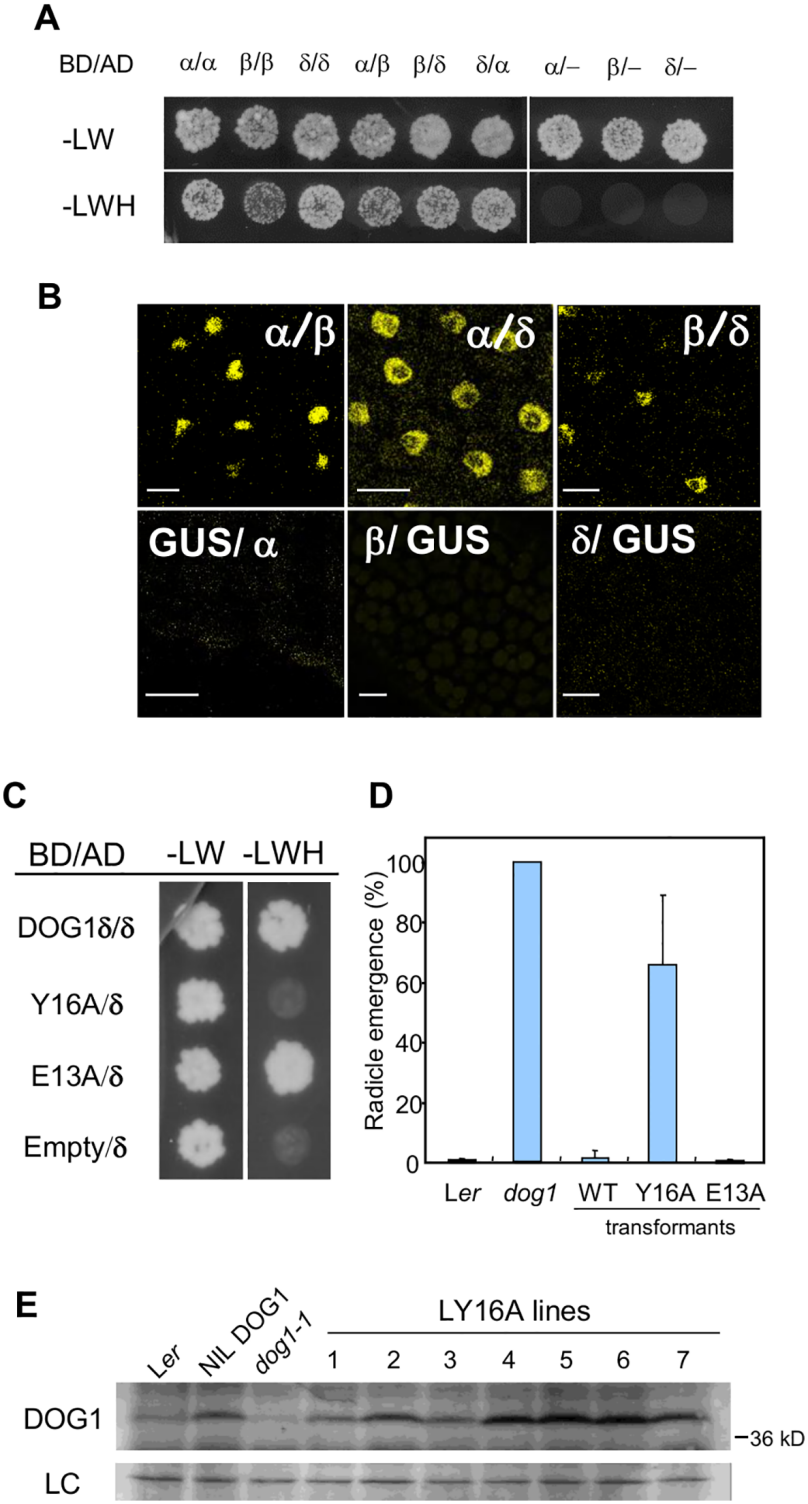
Self-binding of DOG1 affects DOG1 functionality in the seeds. (**A**) Interaction between DOG1 isoforms detected with the yeast two-hybrid assay. BD, fusion with GAL4-DNA binding domain; AD, fusion with GAL4- activation domain; -LW, dropout media without leucine and tryptophan; -LWH, dropout media without leucine, tryptophan and histidine; -, empty vector without DOG1 cDNA. (**B**) Interaction between DOG1 isoforms in planta detected with the split YFP system. Restored YFP fluorescence was observed by confocal microscopy in the embryo of 1-h imbibed seeds. Bar = 10 μm. α/β means the combination of N-terminal half YFP-DOG1 alpha fusion and C-terminal half YFP-DOG1 beta fusion. GUS protein was used as a negative control. (**C**) Interaction between the substituted mutant *dog1* proteins and wild-type DOG1 delta protein. Details are described in the legend of Fig 4A. E13A is shown as a representative of a substitution that does not affect DOG1 binding abilities. (**D**) Germination profiles upon harvest of transgenic lines with alanine-substitutions. The values are the average of several independent lines for Y16A and E13A in *dog1-1*. Error bars represent S.E.M. of at least three biological replicates. (**E**) DOG1 protein accumulation in Y16A transgenic lines. Lines 1 to 7 represent independent transgenic lines. Details are described in the legend of Fig 2F.

Overlapping nuclear localisation and instability of single DOG1 isoforms have prompted us to test mutual binding abilities of the DOG1 isoforms in a yeast two-hybrid experiment. All three DOG1 isoforms were able to bind to themselves and to each other in any combination (Fig 4A). These results were confirmed using the bimolecular fluorescence complementation assay based on split YFP. As shown in Fig 4B, YFP fluorescence could be observed in the nuclei of Arabidopsis embryo cells containing two transgenes consisting of the N- and C- terminal halves of YFP fused to different DOG1 splicing forms. Fluorescence was not detected in the controls.

A series of truncated DOG1 proteins was prepared to identify the region required for self-binding. A yeast two-hybrid assay between these truncated DOG1 proteins and full-length DOG1-δ identified a region of 10 amino acids whose absence makes the protein incapable of binding. Further alanine-scanning experiments in this region revealed that a single replacement, a tyrosine (the 16^th^ amino acid of DOG1_L*er*) with alanine, strongly reduced self-binding (Fig 4C), whereas the other seven obtained substitution mutants did not show altered self-binding abilities.

We were interested whether self-binding is necessary for DOG1 function. To answer this question, a complementation experiment was carried out using two constructs. The first contained the genomic region of the *DOG1* gene from L*er* and the second was identical except for the replacement of tyrosine 16 by alanine (Y16A). Both constructs were transformed into the *dog1-1* mutant and transformants with single insertion events were selected. As shown previously [18], the genomic *DOG1* clone complemented the *dog1* mutant and seeds from the transformants were dormant. In contrast, the Y16A clone showed very weak complementation and 65% of the seeds germinated directly after harvest (Fig 4D and S3 Fig). Therefore, DOG1 protein requires self-binding to induce seed dormancy, although lack of binding ability does not abolish its function completely. A control experiment showed that replacement of glutamic acid at position 13 with alanine, an amino acid that is not affecting self-binding, did not cause a reduced complementation of the *dog1* mutant (Fig 4D and S3 Fig).

Several independent Y16A transgenic lines were further analysed for DOG1 protein accumulation. Interestingly, DOG1 protein could be detected in all of these lines at similar levels as in the dormant NIL DOG1 control (Fig 4E). This indicated that the weak complementation of the Y16A lines was not due to reduced accumulation or instability of the Y16A mutant protein. Because the Y16A-DOG1 protein cannot bind to itself, we concluded that self-binding of DOG1 does not influence its protein accumulation but is required for its full function.

### Differences in DOG1 self-binding ability contribute to natural variation for seed dormancy

*DOG1* was originally identified as a major QTL underlying natural variation in dormancy between the Arabidopsis accessions L*er* and Cvi. Later on, the *DOG1* dormancy QTL was identified in several additional recombinant inbred line populations [28]. This suggests that DOG1 is a major contributor to natural variation for seed dormancy in Arabidopsis. *DOG1* alleles from different accessions show sequence polymorphisms in both promoter and coding regions. A correlation was found between dormancy levels and *DOG1* transcript levels among different genotypes [17]. Accordingly, it is likely that polymorphisms in the promoters of different accessions cause variation in *DOG1* strength between accessions.

**Fig 5:**
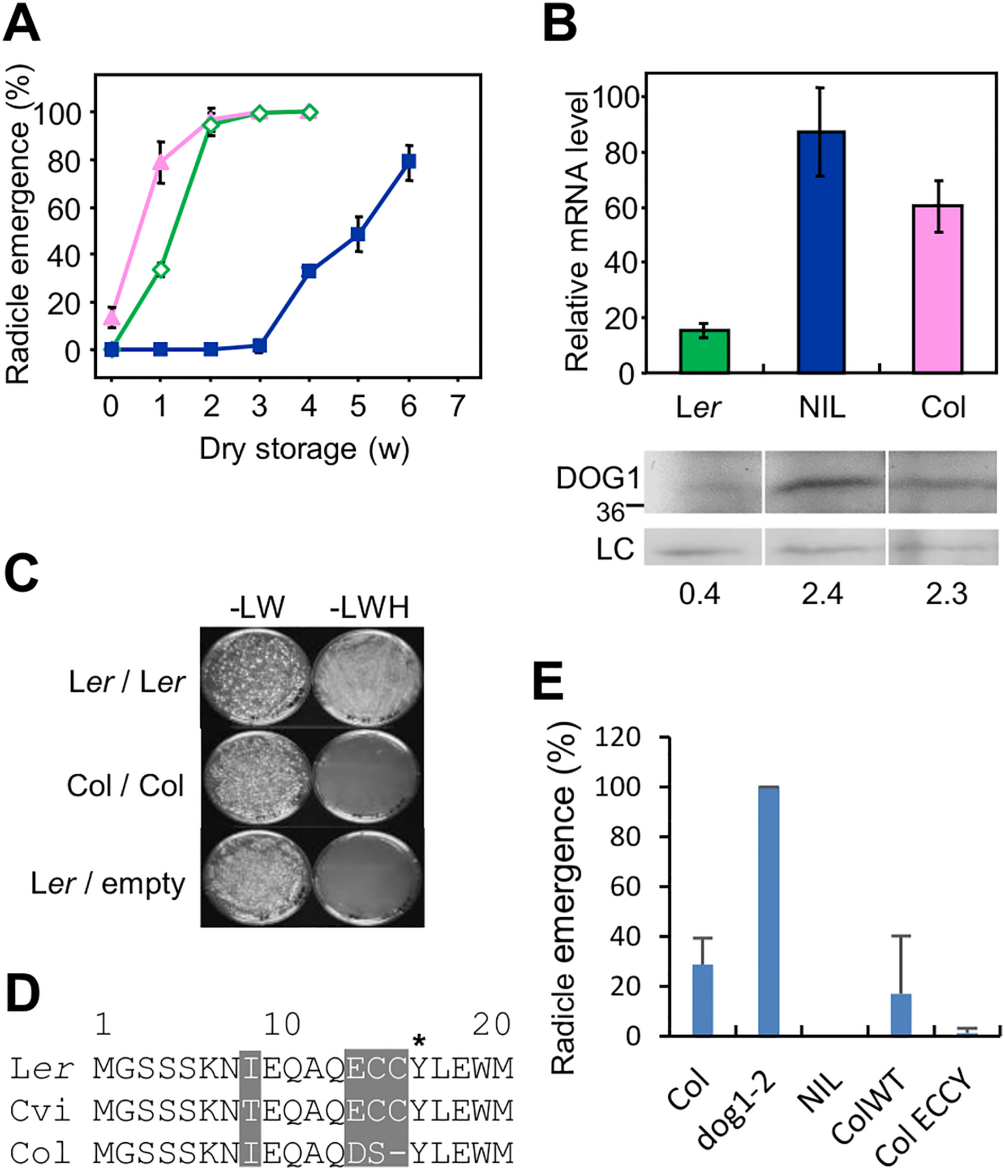
DOG1_Col is a natural non-binding weak allele. (**A**) Germination profiles of Col, L*er* and NIL DOG1 after different periods of dry storage. Pink triangle, Col; green open diamond, L*er*; and blue square, NIL DOG1. Error bars represent S.E.M. of at least three biological replicates. w, week. (**B**) *DOG1* overall mRNA and protein levels in L*er*, NIL DOG1 and Col. Details are described in the legend of Fig 2E and 2F. Numbers below the blot indicate relative DOG1 protein levels, normalized to the loading control. Error bars represent S.E.M. of at least three biological replicates. The data for L*er* and NIL DOG1 were taken from [18] (www.plantcell.org): Copyright American Society of Plant Biologists. (**C**) Yeast two-hybrid binding assay of the beta isoforms of DOG1_Col and controls. Details are described in the legend of Fig 4A. (**D**) Alignment of the first 20 amino acid residues of DOG1 of L*er*, Cvi and Col. The first M is the starting methionine. Polymorphic residues are shaded in grey, and the asterisk represents the 16^th^ tyrosine_L*er* that is required for self-binding. (**E**) The ECCY substitution of DOG1_Col shows enhanced dormancy. Germination profiles upon harvest of transgenic lines with ECCY substitution and controls. Col WT represents 14 independent lines of *dog1-2* transformed with wild-type Col_*DOG1*, Col ECCY represents 13 independent lines of *dog1-2* transformed with the ECCY variant of Col_*DOG1*. Error bars represent S.E.M.

Surprisingly, a comparison of *DOG1* transcript and dormancy levels in the low dormant accessions L*er* and Col, and the dormant line NIL DOG1 (which contains the Cvi allele of *DOG1*) showed that this correlation was absent in Col, which has relatively high *DOG1* transcript levels (Fig 5A and 5B). The DOG1 protein levels of these three genotypes correlated with their transcript levels (Fig 5B) and the Col seeds showed low dormancy despite having high levels of DOG1 protein. This lack of correlation might be caused by a reduced function of the DOG1_Col protein. Therefore, the self-binding ability of DOG1_Col was analysed. As shown in Fig 5C, DOG1_Col showed significantly reduced self-binding in a yeast two-hybrid assay. A sequence comparison of the predicted DOG1 proteins of L*er*, Col and Cvi showed a polymorphism within AA13-16 (Fig 5D). In L*er* and Cvi this region contains the amino acids ECCY, which are replaced by DSY in Col. Interestingly, the tyrosine at AA16 that is required for self-binding is present in all three accessions. Nevertheless, the observed amino acid changes are likely to affect self-binding because the Col DOG1 protein is not able to bind to itself (Fig 5C). To test the influence of this polymorphism on seed dormancy, two constructs containing different *DOG1* alleles were introduced into the *dog1-2* mutant (in Col background) for complementation. One of the constructs contained the wild-type *DOG1*_Col allele, including a 2.2 kb fragment upstream of the START codon and 1.1 kb downstream of the STOP codon. The other construct coded for a modified Col DOG1 protein in which the amino acids DSY at AA13-16 were replaced by ECCY. Transgenic plants containing single introgression events were selected for both constructs and their seed dormancy levels were assessed by following their germination rate during extended seed storage. Similar to the *DOG1* complementation lines previously obtained [18], independent transgenic lines with the same construct showed varying degrees of dormancy (S3 Fig). However, plants with the construct encoding the ECCY Col DOG1 protein showed enhanced dormancy levels compared to plants containing the wild-type Col *DOG1* construct (Fig 5E and S4 Fig). Therefore, we assume that the polymorphism at AA13-16 contributes to the low seed dormancy of Col by reducing the self-binding ability of the DOG1 protein.

**Fig 6:**
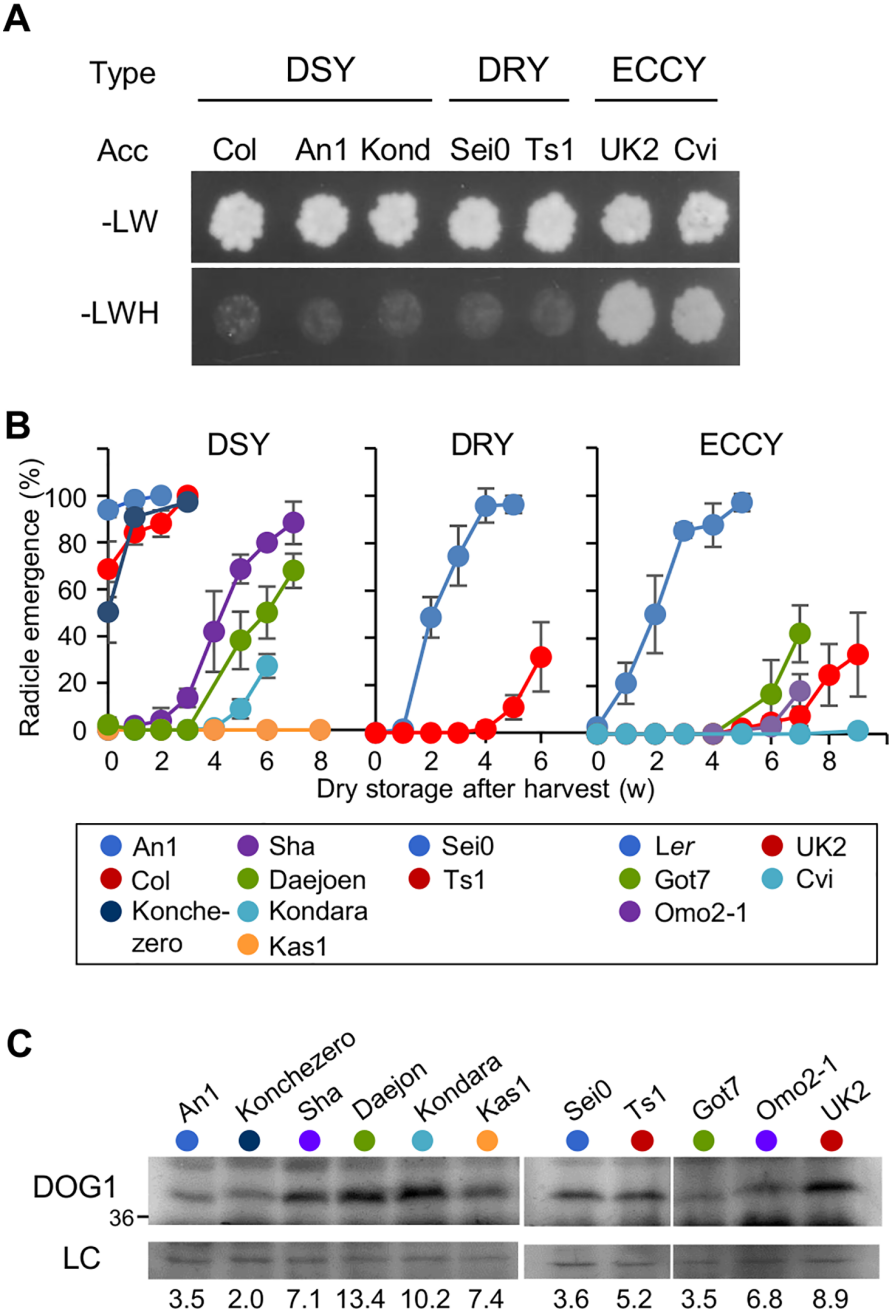
Protein polymorphisms and variation in expression levels of DOG1 both contribute to seed dormancy. (**A**) Interaction of DOG1 beta forms with themselves in different accessions by yeast two-hybrid assay. cDNAs for beta DOG1 were cloned from several accessions of each DOG1 haplotype. Acc, accession; -LW, dropout media without leucine and tryptophan; -LWH, dropout media without leucine, tryptophan and histidine. (**B**) Germination profiles of representative accessions of the three DOG1 haplotypes after different periods of dry storage. Error bars represent S.E.M. of at least three biological replicates. (**C**) DOG1 protein accumulation in accessions. Details are described in the legend of Fig 2F. Numbers below the blot indicate relative DOG1 protein levels, normalised to the loading control.

Natural variation for DOG1 self-binding ability was further explored by analysing DOG1 in 58 accessions. A protein sequence comparison identified three main haplotypes for the AA13-16 region, which were named DSY (Col-type), DRY (Sei0-type) and ECCY (L*er*/Cvi-type) (S5 Fig). Several accessions from each haplotype were analysed for their DOG1 binding ability using a yeast two-hybrid assay. As expected, the haplotype DSY showed very weak self-binding, which was also the case for the DRY haplotype. In contrast, the ECCY accessions showed strong DOG1 self-binding (Fig 6A). We further studied the DOG1 haplotypes by analysis of their dormancy level and DOG1 protein accumulation in fresh seeds of representative accessions of the three groups (Fig 6B and 6C). By combining DOG1 protein levels and haplotype, we could explain a major part of the dormancy levels of these accessions. The DSY accessions had relatively low dormancy levels except for Sha, Daejoen, Kondara and Kas1 that all showed high DOG1 protein levels. Most of the ECCY accessions had high dormancy levels while having low to medium high DOG1 protein levels. The ECCY accession L*er* showed low dormancy, but this accession had very low DOG1 protein levels (Fig 5B). Overall, these data demonstrated that not only expression levels of DOG1 but also differences in self-binding ability affect the strength of DOG1 function in dormancy induction of Arabidopsis natural accessions. We propose that combining the analysis of DOG1 protein levels in freshly harvested seeds with amino acid composition at the 13–16 AA region can lead to an improved prediction of seed dormancy levels in Arabidopsis accessions.

## Discussion

The timing of seed germination determines successful plant establishment. Seed dormancy is a major factor controlling germination potential and has a complex regulation involving several independent pathways [15,28]. The molecular mechanisms that regulate seed dormancy are only beginning to be understood. *DOG1* has been identified as a key dormancy gene in Arabidopsis [17,18]. Seed dormancy levels are well correlated with DOG1 protein levels in freshly matured seeds. The DOG1 protein undergoes modification during dry seed storage, which is paralleled by loss of dormancy [18]. A better understanding of seed dormancy requires a thorough knowledge of the regulation of DOG1. Our present study demonstrated that the accumulation of DOG1 protein is influenced by alternative splicing of the *DOG1* gene. In addition, we have shown that DOG1 protein function is enhanced by its self-binding.

Alternative splicing is widespread in plants, but relatively few studies have been performed to study its regulatory action in specific plant processes. The main consequences of alternative splicing are alteration or loss of protein function, tissue specificity, and changed mRNA stability and turnover via nonsense mediated decay [3,4]. Here, we demonstrated that the production of several protein isoforms by alternative splicing stimulates the accumulation of DOG1 protein. Five *DOG1* splicing variants were identified that are translated into three protein isoforms, DOG1-α, DOG1-β and DOG1-δ. These isoforms could not complement the *dog1* mutant when they were expressed from the endogenous *DOG1* promoter and DOG1 protein accumulation required the presence of at least two of these isoforms. However, overexpression experiments showed that the isoforms had small differences in their functionality with DOG1-β being the most effective, followed by DOG1-δ and DOG1-α (Fig 3). The individual *DOG1* splicing variants and protein isoforms showed a high variation in abundance in wild-type plants. The transcripts encoding the DOG1-β isoform were about 20 times more abundant in mature seeds compared to the transcripts encoding the other two protein isoforms. In accordance, in wild-type seeds DOG1-β showed the highest accumulation and the other isoforms could not be clearly detected on immunoblots. However, our analyses with transgenic plants suggested that the DOG1-β isoform could not sufficiently accumulate in the absence of additional isoforms.

The mechanism that underlies the requirement of multiple isoforms for DOG1 protein to accumulate still needs further characterisation, but we have identified several of its characteristics. First, it is likely that the mechanism depends on active protein degradation because overexpression of single DOG1 isoforms by the 35S promoter can lead to the accumulation of DOG1 protein. We assume that the active DOG1 degradation mechanism is not able to deal with high amounts of single DOG1 isoforms that are continuously translated from abundantly present transcripts. Secondly, the ratio between isoforms appears to be important because equal amounts of splicing forms in the (double and triple) transgenic plants lead to a low level of protein accumulation. The natural ratio between DOG1 isoforms, in which DOG1-β is much more abundant than the other isoforms, leads to high levels of protein accumulation.

*DOG1* is an essential gene for seed dormancy and its protein abundance is correlated with dormancy levels. Therefore, the regulation of DOG1 protein accumulation by alternative splicing could be part of a mechanism to fine-tune seed dormancy. We previously showed that DOG1 protein abundance does not decrease during the last part of the seed maturation phase, although *DOG1* transcript levels are strongly reduced [18]. Interestingly, the *DOG1-δ* transcript variant becomes relatively more abundant at the end of seed maturation in comparison to the variants encoding DOG1-β (Fig 1C). This altered ratio might enhance DOG1 protein accumulation and could explain the persistence of DOG1 protein at this time point, despite a general reduction in *DOG1* transcript levels. Identification of the factors controlling alternative splicing of *DOG1* can lead to a better understanding of its regulation and give new insights in seed dormancy. We have previously identified a splicing factor, SUPPRESSOR OF ABI3 (SUA), which functions during seed maturation and regulates alternative splicing of *ABI3* [10]. We analysed *DOG1* splicing in the *sua* mutant, but did not observe any difference compared to wild-type plants. Therefore, other splicing factors than SUA regulate *DOG1* alternative splicing. One of these has recently been identified as the spliceosome disassembly factor AtNTR1. The *atntr1* mutants have altered transcript levels and splicing of *DOG1* as well as reduced dormancy [27].

Our observation that the accumulation of DOG1 protein requires the presence of multiple isoforms inspired us to analyse whether these isoforms can bind to each other. Indeed, we observed self-binding of DOG1 but contrary to our expectation self-binding was not required for protein accumulation because DOG1 still accumulated in a modified version of DOG1 that is unable to bind to itself (Fig 4). However, this modified version of DOG1 had a strongly reduced function as evidenced by the low dormancy level of seeds that were derived from transgenic plants containing DOG1 that is unable to bind to itself. This observation suggests that DOG1 acts in a protein complex that comprises of at least a homodimer.

*DOG1* has originally been identified as a seed dormancy QTL and the *DOG1* locus shows sequence variation in both promoter and coding region among accessions [17]. It was also shown that variation in *DOG1* expression contributes to variation in seed dormancy levels between Arabidopsis accessions [17]. In this work, we have shown additional natural variation at AA13-16 for DOG1 self-binding. Variation in self-binding ability could contribute to variation in DOG1 function and thereby seed dormancy. As DOG1 is a conserved gene, combining the variation in *DOG1* expression levels with the variation in DOG1 self-binding has the potential to be developed into a marker to predict dormancy levels of crop seeds. However, these two factors are still not enough to fully explain the variation in seed dormancy levels between Arabidopsis accessions. Although *DOG1* is a major dormancy QTL in Arabidopsis, ten other dormancy QTLs have been identified and variation in these QTLs should also be considered. For instance, the high dormancy levels of the Sha and Kondara accessions that have weak non-self-binding *DOG1* alleles might be explained by their strong *DOG6* alleles [28].

In this and previous studies we have observed a strong negative correlation between DOG1 protein levels and germination potential with two important exceptions. Firstly, after-ripened seeds can germinate in the presence of high levels of DOG1 protein [18]. Secondly, we showed in the present work that seeds containing DOG1 that is not able to bind to itself can germinate. We speculate that the lack of a negative correlation between DOG1 protein accumulation and germination potential could have the same cause in both cases. After-ripening has been shown to be associated with DOG1 protein modifications. These protein modifications might prevent or reduce DOG1 self-binding and thereby its function.

## Methods

### Plant materials and growth conditions

*Arabidopsis* NIL DOG1_Cvi is a near isogenic line that contains the *DOG1* allele from Cvi in a L*er* background [18]. The mutant *dog1-1* has been obtained in the NIL DOG1 background [17], *dog1-2* in Col [18]. Arabidopsis accessions used in this study are listed in S1 Table. The double and triple homozygous transgenic lines were selected after crossings using PCR to confirm their homozygosity. All plants were sown on soil and grown in a growth chamber with 16 h- light/8 h-dark cycle (22°C/16°C), or in a greenhouse where the temperature was maintained close to 23°C, 16 h of light was provided daily. Six weeks vernalisation was applied to the late-flowering accessions to promote flowering. Freshly harvested seeds were immediately used for experiments or stored under constant conditions (21°C, 50% humidity, in the dark) for after-ripening treatment.

### Germination tests

About 50 seeds were plated onto a filter paper moistened with demineralized water in Petri dishes and incubated in an alternating condition (12 h light/12 h dark, 25°C/20°C cycle). Radicle emergence was scored after three days, since *dog1-1* mutant and after-ripened seeds of other accessions fully germinate within this period. Each germination test was done in at least three replicates from independent plants.

### RNA extraction and quantitative RT-PCR

Total RNA was extracted from developing *Arabidopsis* siliques as described previously [29]. Quantitative RT-PCR was performed as described previously [18], except for the annealing temperature, which was 64°C for splicing variant-specific primer sets. Sequences of the primers used for qRT-PCR are listed in S2 Table. The expression value for each gene was quantified using a standard curve with a serial dilution of plasmid of known concentration, and they were normalised to the value of *ACT8* (At1g49240) or *HBT* (At2g20000) genes. At least three biological replicates were analysed.

### 3’-RACE

To identify additional splicing variants, 3’-RACE was performed using RNA extracted from L*er* siliques at 16 days after pollination (DAP) following the standard protocol of 3’-Full RACE Core Set (TAKARA) using the oligo-dT-adapter primer, adapter primer and *DOG1* primer 5’-GGATTCTATCTCCGGTACAAGGA- 3’.

### Protein extraction and immunoblot analysis

Seed protein extraction and immunoblot analysis were performed as described previously using peptide antibody against DOG1 [18].

### Construction of transgenic lines

All the binary constructs were prepared using the Gateway technology (Invitrogen). A 5.07 kb fragment of Col genomic DNA corresponding to the L*er* fragment [18] including a 2.22 kb region upstream of the *DOG1* start codon, the *DOG1* coding region and 1.03 kb downstream of the stop codon, as well as cDNA fragments of each splicing variant from Cvi were cloned into pENTR/D-TOPO vector. Entry clones carrying Y16A and E13A mutations in DOG1_L*er* or the ECCY mutation in DOG1_Col were generated by site-directed mutagenesis based on the sequences of DOG1_L*er* using the QuickChange II XL site-directed mutagenesis kit (Stratagene). The resultant genomic entry clones were converted into pGWB1 [30] by LR reaction. For isoform specific complementation, each variant cDNA fragment was cloned under the DOG1 promoter_Cvi into the pGreen backbone. For fluorescent protein fusion constructs, each variant cDNA was cloned into 2x Pro 35S: YFP vectors, pENSG-YFP (N-terminal fusion) and pEXSG-YFP (C-terminal fusion), and split YFP vectors, pBatTL-B-sYFPn and pBatTL-B-sYFPc [31] via LR reaction. All the binary constructs were introduced by electroporation into *Agrobacterium tumefaciens* strains GV3101 or GV3101 carrying the helper plasmid pMP90RK [32] or pSoup [33], which were subsequently used to transform L*er*, *dog1-1* or *dog1-2* plants by floral dipping [34]. All the transgenic lines were first selected based on their antibiotics resistance, their homozygosity was further confirmed by PCR-based genotyping.

### Confocal microscopy analysis

Subcellular localisation of each DOG1 isoform was analysed using binary constructs with single variant cDNA from Cvi fused to YFP at their N-terminus or C-terminus and cloned under the CaMV 35S promoter. Transiently expressed fusion proteins were observed in *Nicotiana benthamiana* leaves as described [35] or in *Arabidopsis thaliana* protoplasts (from Col) as described [10].

For BiFC assays, embryos from 1 h-imbibed seeds of the double homozygous transformants were dissected from testa/endosperm, and restored YFP fluorescence was analysed. Observations were performed with either Zeiss LSM510 or LSM700 confocal laser scanning microscope system using 514 nm lasers for excitation with 63x oil-immersion objective. The images were analysed using the LSM5 software or ZEN imaging software (Zeiss, Germany).

### Yeast-two-hybrid assay

All three cDNA fragments corresponding to the alpha, beta and delta isoforms from L*er*, and the beta isoforms from all other accessions were cloned into pENTR/D-TOPO (Invitrogen), and then recombined in the pACT2-gateway (GAL4 AD fusion) and pAS2-gateway (GAL4 BD fusion) vectors (modified from Clontech). Yeast two-hybrid assays were carried out in yeast strain PJ69-4A [36]. Yeast transformation was performed using a LiAc/SS carrier DNA/PEG method as described [37]. Co-transformed colonies were selected on synthetic dropout medium (SD) lacking Leu (L) and Trp (W). Interaction tests were performed on SD lacking L, W, and His (H) with 5 mM 3-aminotriazole.

### Analysis of DOG1 genomic sequences in Arabidopsis accessions

DOG1 genomic sequences were collected either by Sanger sequencing of PCR-amplified genomic fragments or re-analysis of the publicly available next generation sequencing reads [38]. The Cao’s dataset covered 80 accessions. Additionally, we included data from Col-0, L*er* and Cvi [39].

The polymorphic data are available on Arabidopsis 1001 Genome browser (http://signal.salk.edu/atg1001/3.0/gebrowser.php), however the polymorphisms (SNPs and small INDELs) in the region of interest in L*er* and Cvi (we obtained genomic and cDNA sequences by Sanger sequencing) were not correctly shown in the browser. In order to verify or discover new structural variations, the raw reads were assembled using a program (http://mandrake.mpipz.mpg.de:8081/cgi-bin/oscar.pl) and the contigs were constructed for the DOG1 genomic region in each accession. The obtained DOG1 sequences from accessions that successfully assembled into contigs were aligned to the Col-0 reference. The structural variations of those accessions were used for further analysis.

## Supporting Information

S1 Fig Transcript analysis of *DOG1* splicing variants during seed maturation. (A) qRT-PCR analysis of *DOG1* splicing variants during seed maturation of NIL DOG1. The mRNA level of each *DOG1* variant was normalised to the *HBT* mRNA level. Error bars represent the S.E.M. of at least three biological replicates. (B) The relative ratios of the *DOG1* splicing variants during seed maturation, calculated from the data in (A). The right panel is a magnified image of the bottom of the graph in the left. For clarity, the epsilon variant is not shown in the right panel. Error bars represent S.E.M.
(TIF)

S2 Fig Subcellular localisation of transiently expressed DOG1 isoforms fused to YFP in Arabidopsis protoplasts. Protoplasts were prepared from Arabidopsis young rosette leaves and transient transformation was performed by infiltration using the C-terminal YFP fusion constructs. YFP fluorescence (A–C), autofluorescence of chlorophyll (D-F), and merged image of YFP fluorescence, autofluorescence of chlorophyll, and transmission (G–I). Scale bars = 20 μm.
(TIF)

S3 Fig Germination profiles of independent transformants of DOG1_L*er* substitution lines after different periods of dry storage. L WT, L*er* WT construct; L Y16A, L*er* Y16A substituted line; and L E13A, L*er* E13A substituted line. Bars represent S.E.M. of at least three biological replicates. w, week. L WT data was taken from [18] (www.plantcell.org): Copyright American Society of Plant Biologists.
(TIF)

S4 Fig Germination profiles of independent transformants of Col WT and ECCY substitution after different periods of dry storage. Bars represent S.E.M. from at least three biological replicates. w, week.
(TIF)

S5 Fig Natural variation in the primary amino acid sequence of exon1 of *DOG1*. Genomic or cDNA sequences in the first exon of *DOG1* were determined by either Sanger sequencing of amplified fragments or contig reconstruction from publicly available next generation sequencing (NGS) reads. Deduced amino acid sequences of the first exon are aligned based on the polymorphisms at the 13^th^ E to 16^th^ Y in Ler/Cvi, which is marked by the red open box. The first column shows a list with accessions and the top row the amino acid sequence of the Col accession. Three main haplotypes (DSY, DRY and ECCY) were distinguished based on polymorphisms at amino acids 13–16. Several accessions have missing dots in their sequences due to the ambiguous calls or poor coverage of the region with NGS reads. “.” = identical amino acid; “-” = absent amino acid.
(PDF)

S1 Table Arabidopsis accessions used in this study. (PDF)

S2 Table Sequences of the primers used for qRT-PCR. (PDF)
