# Supplementary figures and images for: Seed Dormancy in Arabidopsis Requires Self-Binding Ability of DOG1 Protein and the Presence of Multiple Isoforms Generated by Alternative Splicing
Source: PLoS Genet. 2015 Dec 18;11(12):e1005737. doi: 10.1371/journal.pgen.1005737 (PMC4686169; doi:10.1371/journal.pgen.1005737)

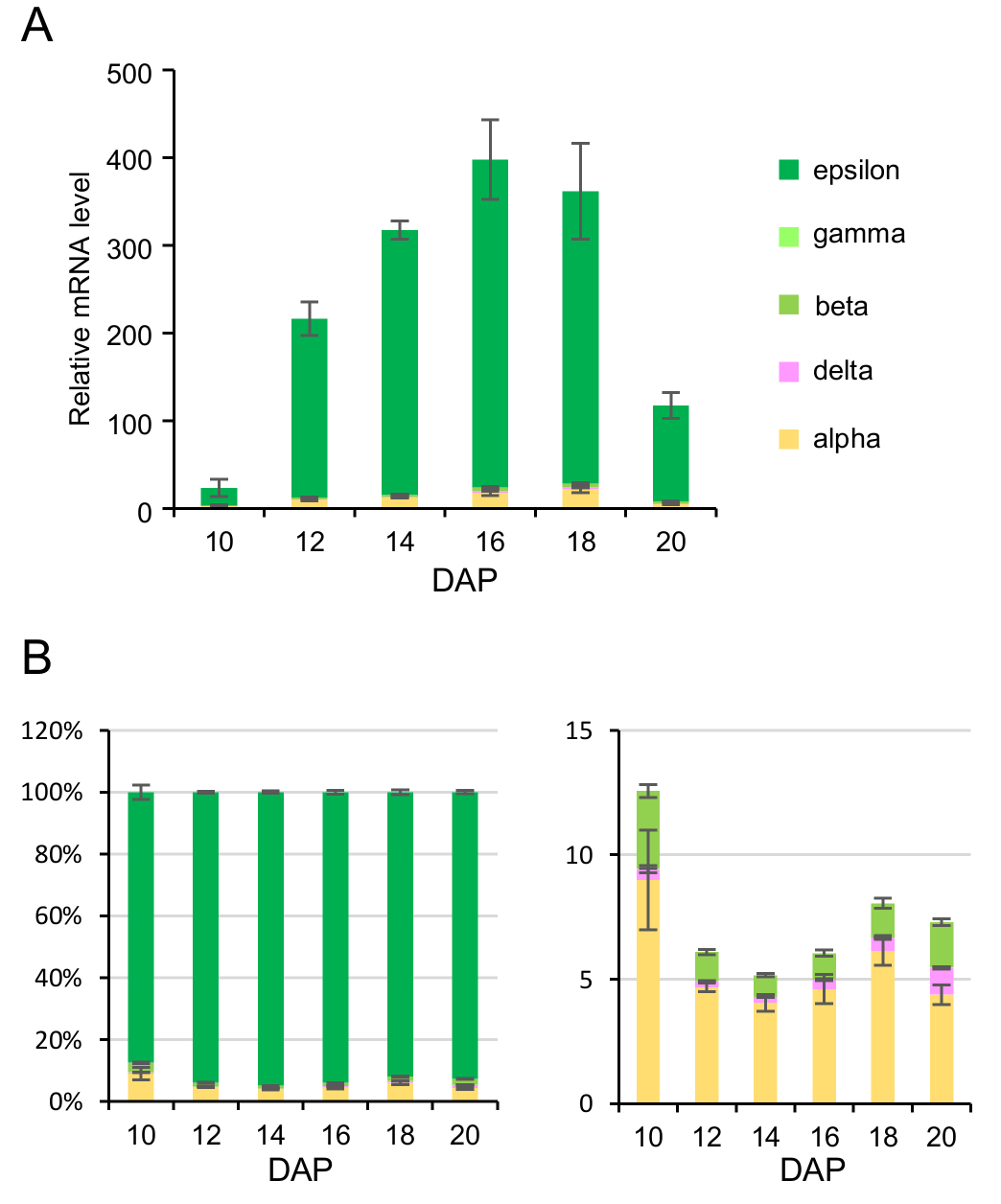

Supplement: S1 Fig — (A) qRT-PCR analysis of DOG1 splicing variants during seed maturation of NIL DOG1. The mRNA level of each DOG1 variant was normalised to the HBT mRNA level. Error bars represent the S.E.M. of at least three biological replicates. (B) The relative ratios of the DOG1 splicing variants during seed maturation, calculated from the data in (A). The right panel is a magnified image of the bottom of the graph in the left. For clarity, the epsilon variant is not shown in the right panel. Error bars represent S.E.M. (TIF) [file pgen.1005737.s001.tif]

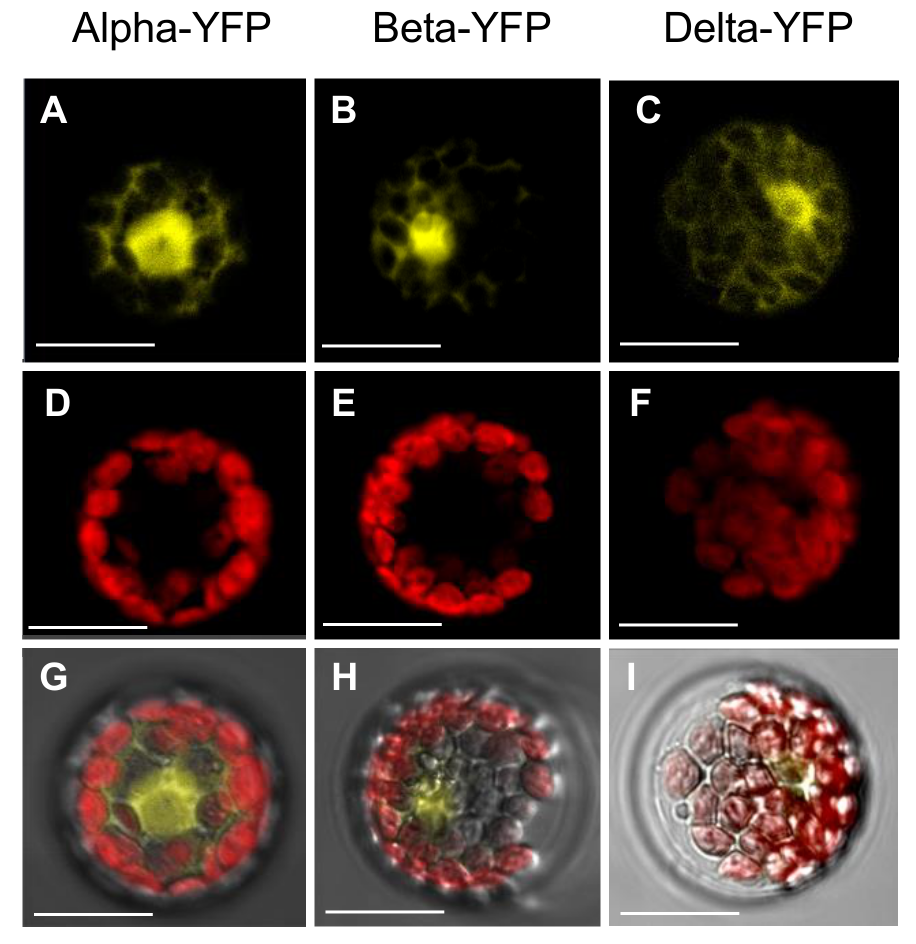

Supplement: S2 Fig — Protoplasts were prepared from Arabidopsis young rosette leaves and transient transformation was performed by infiltration using the C-terminal YFP fusion constructs. YFP fluorescence (A–C), autofluorescence of chlorophyll (D-F), and merged image of YFP fluorescence, autofluorescence of chlorophyll, and transmission (G–I). Scale bars = 20 μm. (TIF) [file pgen.1005737.s002.tif]

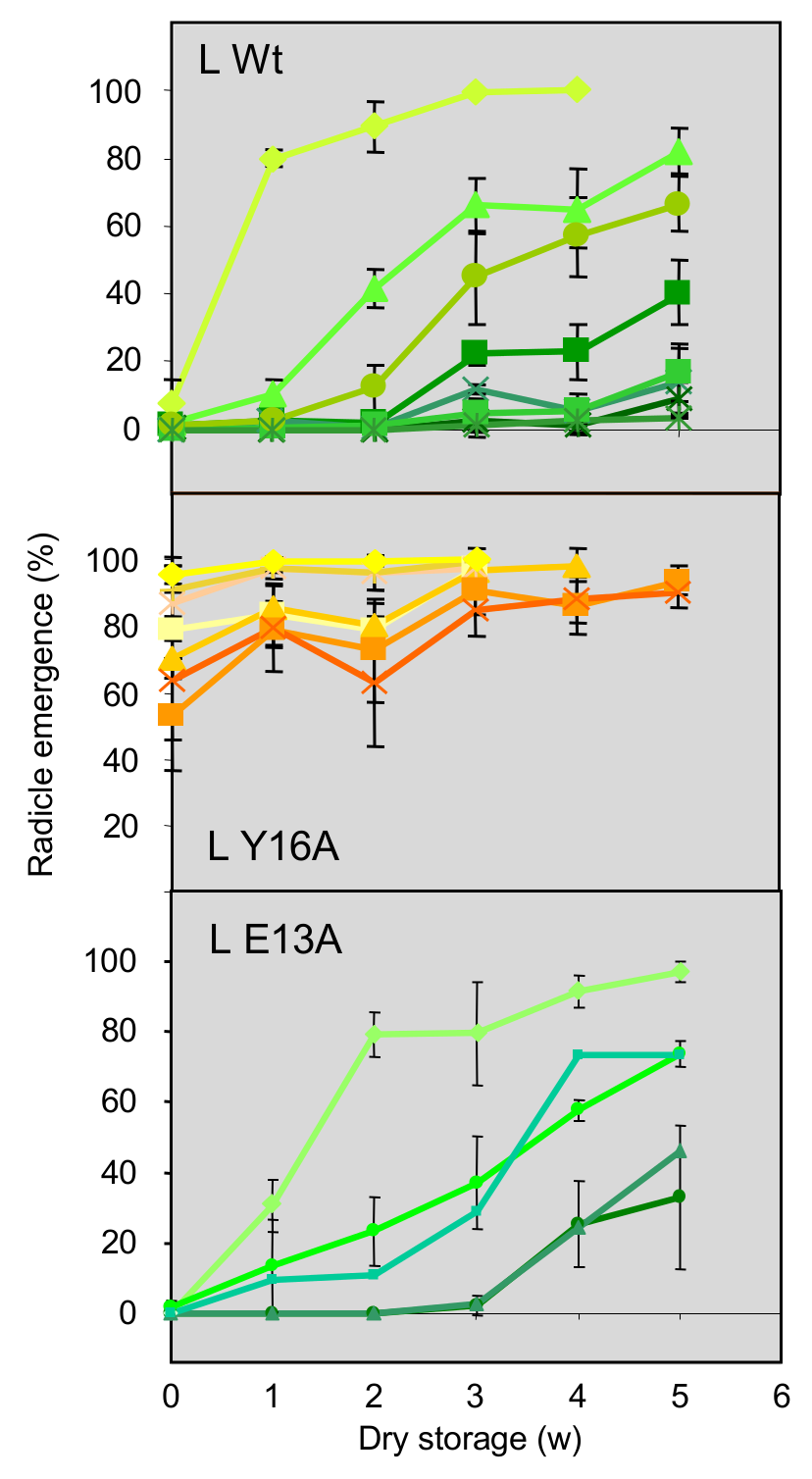

Supplement: S3 Fig — L WT, Ler WT construct; L Y16A, Ler Y16A substituted line; and L E13A, Ler E13A substituted line. Bars represent S.E.M. of at least three biological replicates. w, week. L WT data was taken from [18] (www.plantcell.org): Copyright American Society of Plant Biologists. (TIF) [file pgen.1005737.s003.tif]

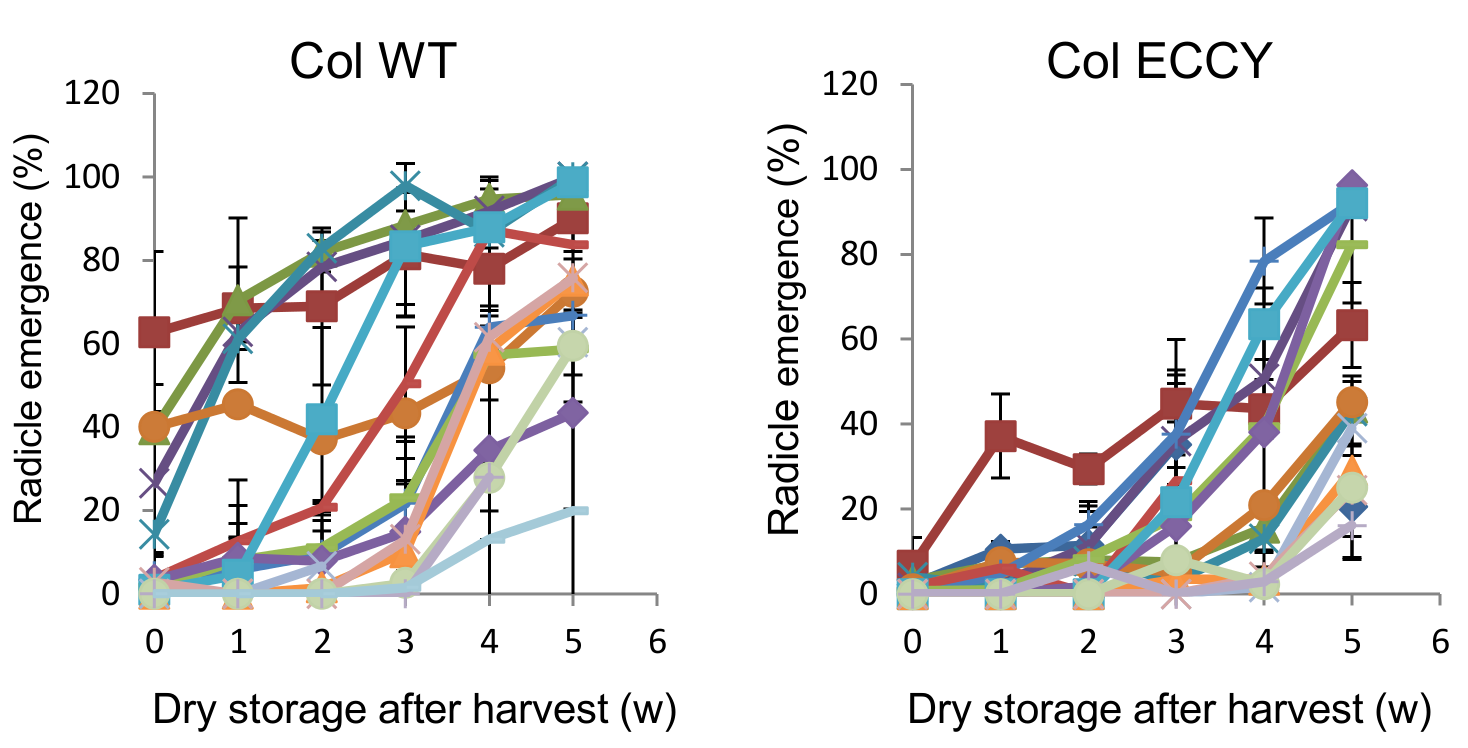

Supplement: S4 Fig — Bars represent S.E.M. from at least three biological replicates. w, week. (TIF) [file pgen.1005737.s004.tif]
