# Supplementary material for: Seed Dormancy in Arabidopsis Requires Self-Binding Ability of DOG1 Protein and the Presence of Multiple Isoforms Generated by Alternative Splicing
Source: PLoS Genet. 2015 Dec 18;11(12):e1005737. doi: 10.1371/journal.pgen.1005737 (PMC4686169; doi:10.1371/journal.pgen.1005737)
Supplement: S5 Fig — Genomic or cDNA sequences in the first exon of DOG1 were determined by either Sanger sequencing of amplified fragments or contig reconstruction from publicly available next generation sequencing (NGS) reads. Deduced amino acid sequences of the first exon are aligned based on the polymorphisms at the 13th E to 16th Y in Ler/Cvi, which is marked by the red open box. The first column shows a list with accessions and the top row the amino acid sequence of the Col accession. Three main haplotypes (DSY, DRY and ECCY) were distinguished based on polymorphisms at amino acids 13–16. Several accessions have missing dots in their sequences due to the ambiguous calls or poor coverage of the region with NGS reads. “.” = identical amino acid; “-” = absent amino acid. (PDF) [file pgen.1005737.s005.pdf]

```
Col          1 MGSSSKNIEQAQDS-YLEWMSLQSQRIPELKQLLAQRRSHGDEDNDNKLRKLTGKIIGDFKNYAAKRADLAHRCSSNYYAPTWNSPLENALIWMGGCRPSSFFRLVYALCGSQTEIRVTQFLRNIDGYESS  130
An-1         1 ..............-...................................E................................................................................  130
C24          1 ..............-...................................E................................................................................  130
Kondara      1 ..............-....................................................................................................................  130
Sha          1 ..............-....................................................................................................................  130
Eden1        1 ..............-....................................................................................................................  130
Konchezero   1 ..............-........................................................................  86
Kas1         1 ..............-...........................................................................  89
HKT2.4       1 ..............-....................................................................................................................  130
Daejeon      1 ..............-...........................................................................  89
Rue3-1-31    1 ..............-................................................................................................................  126
ICE1         1 ..............-....................................................................................................................  130
ICE29        1 ..............-....................................................................................................................  130
ICE61        1 ..............-....................................................................................................................  130
ICE63        1 ..............-....................................................................................................................  130
ICE79        1 ..............-...................................E................................................................................  130
ICE98        1 ..............-...................................E................................................................................  130
ICE106       1 ..............-...................................E................................................................................  130
ICE107       1 ..............-...................................E................................................................................  130
ICE119       1 ..............-...................................E................................................................................  130
ICE120       1 ..............-...................................E................................................................................  130
ICE134       1 ..............-........................................................................  86
ICE212       1 ..............-...................................E...............................................................  113
ICE213       1 ..............-...................................E................................................................................  130
Cdm-0        1 .............R-...................................E................................................................................  130
Sei-0        1 .............R-...................................E................................................................................  130
Ts-1         1 .............R-...................................E................................................................................  130
Mer-6        1 .............R-...................................E................................................................................  130
TueSB30-3    1 .............R-...................................E..............................................................S.................  130
ICE163       1 .............R-...................................E................................................................................  130
ICE169       1 .............R-...................................E................................................................................  130
ICE173       1 .............R-...................................E................................................................................  130
ICE216       1 .............R-...................................E........................................................  106
Agu-1        1 ............ECC...................................E................................................................................  131
Bak-7        1 ............ECC...................................E.........................................................  108
Del-10       1 ............ECCH..................................E................................................................................  131
Ler          1 ............ECC...................................E................................................................................  131
Cvi          1 .......T....ECC...................................E................................................................................  131
Got7         1 ............ECC...................................E................................................................................  131
NES1         1 ............ECC...................................E................................................................................  131
Omo2-1       1 ............ECC...................................E.......................................  90
Fei0         1 ............ECC................P..................E.....................................................F..........................  131
Sah-0        1 .......T....ECC...................................E................................................................................  131
UK2          1 ............ECC...................................E................................................................................  131
Istisu-1     1 ............ECCH..................................E.....................................K...........Y..............................  131
Ped-0        1 ............ECCH..................................E................  67
TueWa1-2     1 ............ECC................P..................E.....................................................F..........................  131
Vash-1       1  ...........ECC...................................E................................................................................  130
Vie-0        1 ............ECC...................................E................................................................................  131
ICE33        1 ............ECC...................................E................................................................................  131
ICE36        1 ............ECC...................................E................................................................................  131
ICE49        1 ............ECC...................................E................................................................................  131
ICE50        1 ............ECC..............................  45
ICE71        1 ............ECC...................................E......................  73
ICE93        1 ............ECC...................................E................................................................................  131
Don-0        1 ............ECS...............................S...E................................................................................  131
Qui-0        1 ............ECS...............................S...E..................................................L......................  124
ICE21        1 .............C-...................................E................................................................................  130
```

DSY (Col type)

DRY (Sei-0 type)

ECCY (Ler/Cvi type)

ECSY

DCY

S5 Fig. Natural variation in primary amino acid sequences in the exon1 of *DOG1*.
