# Supplementary material for: Seed Dormancy in Arabidopsis Requires Self-Binding Ability of DOG1 Protein and the Presence of Multiple Isoforms Generated by Alternative Splicing
Source: PLoS Genet. 2015 Dec 18;11(12):e1005737. doi: 10.1371/journal.pgen.1005737 (PMC4686169; doi:10.1371/journal.pgen.1005737)
Supplement: S1 Table — (PDF) [file pgen.1005737.s006.pdf]

S1 Table. Arabidopsis accessions used in this study.

Accession name	Stock number	Comment
L*er*	CS20	
Col-0	CS1092	
An-1	CS944	
Cvi-1	CS8580	
Deajoen	19206	From Dr. Lee in Korea via Prof. M. Koornneef
Got-7	CS22608	
Kas-1	CS903	
Konchezero	CS22491	
Kondara	CS22651	
Omo2-1	CS22584	
Sei-0	CS1504	
Shakdara	CS929	
Ts-1	CS1552	
Uk-2	CS1578/SJA24000	
