# Supplementary material for: Seed Dormancy in Arabidopsis Requires Self-Binding Ability of DOG1 Protein and the Presence of Multiple Isoforms Generated by Alternative Splicing
Source: PLoS Genet. 2015 Dec 18;11(12):e1005737. doi: 10.1371/journal.pgen.1005737 (PMC4686169; doi:10.1371/journal.pgen.1005737)
Supplement: S2 Table — (PDF) [file pgen.1005737.s007.pdf]

S2 Table. Sequences of the primers used for qRT-PCR.

Gene name	AGI	Primer name	Primer sequences
Actin 8	At1g49240	Actin8-qF	ctcaggtattgcagaccgtatgag
		Actin8-qR	ctggacctgcttcatcatactctg
Hobbit	At2g20000	Hbt-qF	ACAAGACACTACAACGCATGGTAC
		Hbt-qR	TCTCTAGTGCTTCCTCACTTCTCTTC
DOG1	At5g45830	Overall-qF	GAGCTGATCTTGCTCACCGATGTAG
		Overall-qR	CCGCCACCACCTGAAGATTCGTAG
		Variant-qF	GGATTCTATCTCCGGTACAAGGAGCGGATTTC
		Alpha-qR	CCACTATTCACAGTTGTACATGCATCGAATATTACTTC
		Beta-qR	CCACTATTCACAGTTGTACATGCATCGAATATTACTATAG
		Gamma-qR	CGCAAAATGCCACGACGTGAATAAACTATAG
		Delta-qR	CGCAAAATGCCACGACGTGAATAAACTTC
		Epsilon-qR	CACATACGGGTTATATATCCCTAGAATATATGCCA
